# What every intensivist should know about Big Data and targeted machine learning in the intensive care unit

**DOI:** 10.5935/0103-507X.20190069

**Published:** 2019

**Authors:** Ményssa Cherifa, Romain Pirracchio

**Affiliations:** 1 Institut National de la Santé et de la Recherche Médicale - Paris, France.; 2 Université de Paris - Paris, France.; 3 The ACTERREA Research Group - Paris, France.; 4 Department of Anesthesia and Perioperative Medicine, Zuckerberg San Francisco General Hospital, University of California San Francisco - California, United States.; 5 Department of Anesthesia and Intensive Care Medicine, Hôpital Européen Georges Pompidou - Paris, France.

## The increasing importance of Big Data in healthcare

The conjunction of increasingly available access to big medical data and of substantial progress in machine learning (ML) and artificial intelligence (AI) has created new, unforeseen opportunities for data science in healthcare. Big Data is described as having at least three distinct characteristics, volume, velocity, and variety, but in regard to healthcare, it also includes variability and value.^(1)^ Therefore, it is very challenging to extract any useful information from Big Data using traditional statistical methods.^(2)^ Big Data analytics has immense potential for improving quality of care, helping physicians and nurses to make more personalized clinical decisions, reducing waste and errors and possibly reducing the cost of care.^(3)^ Anticipating organ dysfunction before it occurs can be extremely helpful to (i) make better and more tailored therapeutic decisions and (ii) in some instances, prevent the occurrence of organ failure by appropriately adjusting the therapeutics upfront. Additionally, the ability to predict any upcoming deterioration can be very helpful to assist clinical leadership in proactively allocating human resources. Malak et al. recently proposed a multiagent risk management architecture based on Big Data and analytics in order to create a collaborative and real-time environment to manage neonates with critical conditions at the neonatal intensive care unit (ICU).^(4)^

## Sources of healthcare Big Data

The "data revolution" in healthcare and, ultimately, in critical care depends on the ability to stream and store a large amount of information in a protected and encrypted central repository. Electronic medical records, bedside monitors, drug delivery devices, ventilators or dialysis machines are continuously generating data. It is becoming possible to combine these data with laboratory test results, procedures, caregiver notes, imaging reports, and, ultimately, outcomes, including long-term functional and behavioral outcomes. For instance, the Mayo Clinic has developed such a data warehouse, called the (Multidisciplinary Epidemiology and Translational Research in Intensive Care Data Mart (METRIC),^(5)^ while the Beth Israel Deaconess Medical Center (BIDMC) has launched a similar large database comprising deidentified health-related data associated with over 40,000 patients who stayed in critical care units, the MIMIC-III.^(6)^ These two databases are openly available for scientific research purposes. Increasingly more ICUs, medical centers and even large-scale health networks are developing solutions to store and analyze patient data and benchmarks with different systems and organizations.^(7)^

## Machine learning for predictive analytics and decision support in the intensive care unit

**Figure 1:**
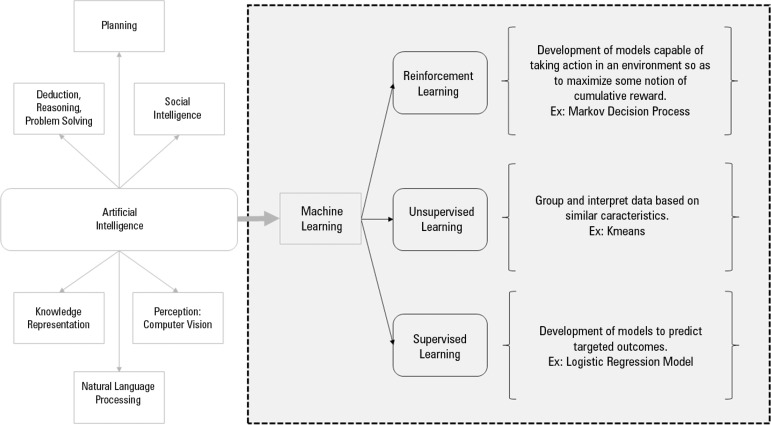
Artificial intelligence and different types of machine learning algorithms.

Because "Big Data includes heterogeneous, multispectral, incomplete and imprecise observations derived from different sources",^(8)^ the development of appropriate analytics and inference is needed. Machine learning, which is the component of AI that allows computers to make data-driven choices and predictions, is now considered as the solution of choice to harness big medical data.^(9)^ Obviously, ML has the ability to model complex relationships between large explanatory features and desired outputs, such as patient outcomes. ML algorithms are usually divided into different categories: parametric vs. nonparametric methods, supervised vs. unsupervised algorithms, and unique vs. ensemble algorithms (Figure 1). Supervised learning algorithms are used to uncover the relationship between potential explanatory features and one or more known target outcomes. They are commonly applied in critical care for the prediction of clinical events, such as the prediction of ICU mortality.^(10)^ In unsupervised learning algorithms, there is no specific targeted outcome; the goal is essentially to dig deep into the data structure in order to identify the correlation between features and create clusters of characteristics. These algorithms are currently mainly used in precision medicine, in which the goal is to uncover subgroups of patients who share similar clinical or molecular characteristics.^(11)^

## Perspectives

The Food and Drug Administration (FDA) describes precision medicine as providing "the right patient with the right drug at the right dose at the right time".^(12)^ With the development of new ML algorithms, it is becoming feasible in the foreseeable future to analyze in real-time gigantic amounts of data directly streamed from the bedside in order to provide more personalized and relevant predictions. This field of stream analytics in which data are collected and used sequentially to update the current predicted algorithms is referred to as online ML.^(7)^ Such an automated technology that is deployable bedside is the path for the ultimate goal of precision medicine. Thus, the next challenges are to create real-time support tools for personalized decision-making, allowing the clinician to better adapt his therapy for patients in critical situations. This current approach, called prescriptive analytics, refers to the prediction of treatment effects at the patient level. A statistical approach derived from causal inference methods may be used to estimate the benefit of treatment at the individual level rather than the population level. The definition and estimation of such parameters will allow, if coupled with Big Data, to support the clinician in his decisions by highlighting optimal therapeutic choice strategies. Komorowski et al. developed a computational model able to dynamically suggest optimal treatments for adult patients with sepsis in the ICU.^(13)^

## Current limitations and conclusions

One needs to acknowledge the existence of limitations that will need to be overcome in order to allow for targeted ML to become a reality in the future.^(14)^ First, while ICUs are now generating gigabytes of data each day, only a small fraction is currently accessible for research purposes.^(15)^ Second, important questions remain about how best to leverage big medical data and ML in the ICU. Randomized controlled trials will be needed to demonstrate the benefit of predictive and prescriptive analytics in critically ill patients. However, considering recent advances, big medical data and ML offer a unique opportunity to dramatically change our paradigm from the era of evidence-based medicine in which therapeutic decisions are essentially based on population-level shreds of evidence to a new era of optimal and personalized clinical decision support.
